# Treatment of everolimus-resistant metastatic renal cell carcinoma with VEGF-targeted therapies

**DOI:** 10.1038/bjc.2011.389

**Published:** 2011-10-27

**Authors:** V Grünwald, C Seidel, M Fenner, A Ganser, J Busch, S Weikert

**Affiliations:** 1 Hannover Medical School, Department of Hematology, Hemostasis, Oncology and Stem Cell Transplantation, Hannover, Germany; 2 Department of Urology, Charite Universitätsmedizin, Berlin, Germany

**Keywords:** tyrosine kinase inhibitor, refractory, sunitinib, sorafenib, everolimus, renal cell carcinoma

## Abstract

**Background:**

Treatment of everolimus-resistant disease remains largely undefined in metastatic renal cell carcinoma (mRCC). We report on 40 patients (pts) who receive systemic treatment after failure of everolimus.

**Patients and methods:**

Forty pts received sunitinib (*n*=19), sorafenib (*n*=8), dovitinib (*n*=10) or bevacizumab/interferon (*n*=3) after failure of everolimus. Median progression-free survival (PFS), overall survival (OS) and best tumour response (according to Response Evaluation Criteria In Solid Tumors) were analysed retrospectively. Kaplan–Meier, log-rank test and Cox regression analyses were used to estimate or predict OS and PFS.

**Results:**

Treatment of everolimus-resistant disease was associated with a PFS of 5.5 months. (range 0.4–22.3) and an objective partial remission (PR) in 4 pts (10%) and stable disease (SD) in 22 pts (55%). In univariate analyses, first-line treatment with sorafenib was the only variable to correlate with a prolonged PFS of treatment in everolimus-resistant disease (*P*=0.036). However, its significance as a predictive marker for subsequent therapy could not be verified in multivariate analyses.

**Conclusions:**

Vascular endothelial growth factor targeted therapy shows promising activity in everolimus-resistant metastatic renal cancer and warrants further studies.

Current targeted therapies in patients (pts) with metastatic renal cell carcinoma (mRCC) consist of inhibitors targeting the vascular endothelial growth factor (VEGF), VEGF receptor or mammalian target of rapamycin (mTOR). Current first-line options in Europe consist of sunitinib, pazopanib and bevacizumab/interferon (Bev/IFN) with a progression-free survival (PFS) of 11, 11 and 10 months, respectively (Escudier *et al*, 2007; Motzer *et al*, 2007; Sternberg *et al*, 2010). The mTOR inhibitor (mTORi) temsirolimus has been approved for first-line treatment of patients at poor risk associated with a PFS of 3.8 months (Hudes *et al*, 2007), and more recently the mTORi everolimus showed to attain a PFS of 4.9 months in pts who failed at least one prior TKI (Motzer *et al*, 2008).

So far only few data are available supporting the use of another treatment line beyond the failure of everolimus. We, therefore, analysed the efficacy of subsequent therapy in pts with mRCC who failed treatment with both TKI and everolimus.

## Patients and methods

Out of 280 mRCC pts treated between November 2005 and November 2010, 78 pts received everolimus either within the phase III trial RECORD 1 (CRAD001C2240; *n*=12) or within RAD001 expanded access programme ‘REACT’ (CRAD001L2401; *n*=40). In 26 pts, everolimus was prescribed as a standard of care. Of these, 40 pts received subsequent treatment after everolimus and were evaluable for our study.

Medical records were retrieved and analysed retrospectively in accordance with the regulatory agreement of the local ethics committee and the Declaration of Helsinki Principles.

Treatment outcome was defined by either median overall survival (OS), which has been calculated from the start of everolimus treatment and median PFS of post-everolimus therapy. Tumour response was evaluated according to the Response Evaluation Criteria In Solid Tumors (RECIST) version 1.0 (Therasse *et al*, 2000). Stable disease (SD), partial remission (PR) or complete remission (CR) was considered as disease control (DC). To detect factors associated with the OS from the beginning of everolimus treatment and the PFS of post-everolimus treatment, univariate and multivariate analyses were performed.

### Patients’ characteristics

Before the use of first-line VEGF-targeted therapy 19 pts (48%) had intermediate and 9 pts (23%) had low risk according to the Memorial Sloan–Kettering Cancer Centre (MSKCC) risk stratification (Motzer *et al*, 2002). Before the use of subsequent VEGF-targeted therapy in everolimus-resistant disease, 3 pts (8%) were at low, 14 pts (35%) were at intermediate and 11 pts (28%) were at high risk according to the MSKCC risk stratification. Further characteristics of pts are shown in detail in Table 1.

### Treatment regimens

Before the use of everolimus every patient received VEGF-targeted therapy.

First-line treatment consisted of sunitinib in 30 (75%), sorafenib in 9 (23%) and Bev/IFN in 1 pt. (3%). Ten pts received another second-line VEGF-targeted therapy. All pts received everolimus and subsequent therapy with sunitinib (*n*=19; 48%), sorafenib (*n*=8; 20%), Bev/IFN (*n*=3; 8%) or dovitinib (*n*=10; 25%); an investigational TKI was given within the CTKI258A2107 protocol (Angevin *et al*, 2010). In two pts. tumour response was not available because of concomitant disease, which led to permanent treatment interruption or loss to follow-up, respectively. Cytokine therapy was applied before first-line VEGF-targeted therapy in 60% of the pts.

Everolimus was given continuously 10 mg OD. Sunitinib was given as 50 mg OD for 28 consecutive days of a given 6-week cycle. Sorafenib was administered continuously at 400 mg b.i.d. Bevacizumab was infused at 10 mg kg^−1^ intravenously every 2 weeks, with IFN applied subcutaneously thrice a week with 9 Mio units. Dovitinib was administered at 500 mg OD in a 5 days on/2 days off treatment cycle.

In case of significant toxicity in sunitinib-treated patients, doses were reduced to 37.5 mg and further to 25 mg OD if necessary. Sorafenib dose reduction consisted of a decrease to 200 mg b.i.d. Dovitinib dose could be reduced to 450  mg and further to 400 mg, respectively.

First-line treatment was given until disease progression in 34 pts (85%), and in 6 pts treatment was stopped because of intolerable toxicity. In two pts, first-line treatment was stopped before the first disease evaluation and in another pt. response to first-line treatment was not evaluable. They were not considered for PFS calculation.

In two pts. no tumour evaluation was performed for subsequent therapy after everolimus. One pt. had a stroke during systemic treatment, whereas one pt. was lost to follow-up. Another pt. developed cerebral metastases shortly after treatment initiation. These pts were not considered for PFS calculation.

### Statistics

Progression-free survival was defined as the time from initiation of subsequent treatment after everolimus to the day tumour progression was proven or death occurred. Patients were censored at the date of last follow-up. Overall survival was defined as the time from initiation of treatment with everolimus to the time of death or censored at the date of last follow-up. Kaplan–Meier curves comparing PFS and OS between patient groups were constructed, and log-rank testing was used to compare these censored outcomes. Multivariate analyses were performed by Cox regression analyses. The following patient characteristics were tested: gender, age, prior cytokine treatment, first-line responder *vs* non-responder, drugs applied as first-line treatment, first-line treatment PFS and best response, duration between first-line treatment and subsequent treatment after everolimus, organ sites affected with metastases, MSKCC score at first-line VEGF-targeted therapy and the Heng score (Heng *et al*, 2009). Calculations were performed using the Statistical Package for the Social Sciences (SPSS) 18 (Chicago, IL, USA).

## Results

### First-line VEGF-targeted treatment

First-line VEGF-targeted therapy was associated with a median PFS of 11.3 months (range 2.2–37.6). Objective tumour response consisted of SD in 14 pts (35%), PR in 12 pts (30%) and a CR in 3 pts (7%), whereas 8 pts (20%) had PD with the first tumour evaluation. In two pts, first-line treatment was interrupted because of toxicity before tumour evaluation and in one pt. response according to first-line VEGF-targeted therapy remains unclear.

Another line of TKI treatment was given in a subset of 10 pts., which provided a median PFS of 5.5 months (range 2–12.7). The median OS from first-line VEGF-targeted therapy is 37.3 months (range 9.6–64.7).

### Everolimus treatment

Patients received subsequent therapy with everolimus either in the second (*n*=30; 75%) or in the third line (*n*=10; 25%). Treatment was associated with a median PFS of 5.9 months (range 1.7–16.7) with SD in 22 pts (55%) and a PR in 2 pts (5%). Twelve pts. had PD with first tumour evaluation. Dose reduction during everolimus treatment was required in three cases due to pneumonitis (*n*=2) or stomatitis (*n*=1). Four pts. were not further evaluated because of treatment intolerance with allergic reaction and dyspnoea (*n*=2) or because of pt. withdrawal (*n*=2).

### Subsequent treatment after everolimus failure

Subsequent treatment after everolimus was associated with SD in 22 pts (55%) and a PR in 4 pts (10%). Eleven pts had PD (28%). The median PFS on therapy after everolimus failure is 5.5 months (range 0.4–22.3), which was associated with a median OS of 11.3 months (range 0.8–22.3) from the commencement of subsequent therapy after failure of everolimus. At the last follow-up, 16 pts were alive, out of whom 6 (23%) remained on the same subsequent therapy after everolimus and 5 pts were on another active treatment. Five pts received the best supportive care.

The median interval between last VEGF-targeted therapy and subsequent therapy after everolimus is 8.4 months (range 0.8–18.9).

### Prognostic and predictive factors

On univariate analyses, treatment with sorafenib was associated with a prolonged PFS after everolimus of 3.7 *vs* 11.3 months (*P*=0.036). On multivariate analyses none of the tested factors were found to be predictive for the PFS of subsequent therapy after everolimus.

A PFS of ⩾6 months during first-line VEGF-targeted therapy was associated with a prolonged OS from first-line treatment on univariate (19.3 *vs* 53.4 months, *P*<0.001) and on multivariate analyses (*P*=0.002; HR 0.08; 95% CI 0.17–0.383; Tables 2, 3, 4 and 5).

## Discussion

In VEGF-resistant disease, treatment with the mTORi everolimus was associated with a median PFS of 4.9 months. The clinical relevance of mTOR inhibition in VEGF-resistant disease is further supported by retrospective analyses reporting a PFS of 3.7–5.1 months for temsirolimus (Schwandt *et al*, 2009; Weikert *et al*, 2010).

The evidence of successful treatment after failure of an mTORi is rare. Recently the response of 13 pts receiving sunitinib re-challenge after the use of mTORi is reported, giving a median PFS of 21 months in first line. At the time of re-exposure, 12 of the 13 pts (92%) again showed clinical benefit that was associated with a median PFS of 6.9 months (Grünwald *et al*, 2011). Another series reported recently on the activity of sorafenib after failure of either temsirolimus or everolimus in 34 pts. Subsequent therapy achieved a median PFS of 4 months and a DCR of 44%, indicating anti-tumour activity of sorafenib after the use of an mTORi (Di Lorenzo *et al*, 2010).

On the basis of these data it seems suggestive that VEGF resistance remains transient in nature, at least in initially susceptible pts.

Our current retrospective analyses aimed to explore the role of subsequent therapy in everolimus-resistant disease and the impact of predictive factors on clinical outcome in these pts. Therapy after failure of everolimus consisted of different inhibitors of the VEGF or VEGFR, and was associated in 65% pts with DC, achieving a PFS of 5.5 months and a median OS of 11.28 months from the commencement of treatment after everolimus. These results indicate that subsequent treatment in everolimus-resistant disease is clinically effective and VEGF resistance may remain transient during the course of treatment.

On multivariate analyses, PFS of first-line VEGF-targeted therapy showed prognostic value in our cohort of pts (*P*=0.002; HR 0.08; 95% CI 0.017–0.383). A recent report by Heng *et al* (2011) also supports the notion that PFS of first-line VEGF-targeted therapy is a strong prognostic factor for the OS of RCC patients. We could confirm these findings in a separate set of 119 pts as a single-center experience, which support the prognostic value of first-line PFS in RCC pts (Seidel *et al*, 2011).

In summary, current treatment algorithms include everolimus as the standard therapy in VEGF-resistant disease. Our data suggest clinical activity of treatment with VEGF-targeted therapies beyond everolimus and spur the notion that VEGF resistance remains transient at least in a subgroup of patients. Several ongoing clinical trials are based on this observation and will test this hypothesis in a phase III setting.

## Figures and Tables

**Table 1:** Patients’ characteristics before the commencement of first-line VEGF-targeted therapy

	***N***	**%**
*Gender*		
Female	14	35
Male	26	65
		
*Median age*		
Years	59	
Range	29–75	
		
*Histology*		
Clear cell	38	95
Papillary	1	3
Sarcomatoid	1	3
		
*Metastatic organs*		
Lung	34	
Liver	15	
Bone	15	
		
*Number of metastatic sites*		
1–2	11	28
3–4	15	38
⩾5	14	35
		
*ECOG*		
0	20	50
1	18	45
2	2	5
		
*MSKCC*		
Low	9	23
Intermediate	19	48
Poor	0	
NA	12	30
		
*Heng prognostic score*		
Low	6	15
Intermediate	17	43
Poor	1	3
NA	16	40
		
*First-line therapy*		
Sunitinib	30	75
Sorafenib	9	23
Bev/IFN	1	3
		
*Response to first-line VEGF-targeted therapy*		
Acquired resistance	29	73
Intrinsic resistance	8	20
Not evaluable	3	8
		
Prior immunotherapy	24	60

Abbreviations: Bev=bevacizumab; ECOG=Eastern Cooperative Oncology Group; IFN=interferon; MSKCC=Memorial Sloan–Kettering Cancer Centre; NA=not available; VEGF=vascular endothelial growth factor.

**Table 2:** Univariable analyses for progression-free survival of subsequent therapy after failure of everolimus

**Independent variables**		**PFS of subsequent therapy in everolimus-resistant disease (in months)**	***P*-value**
Sex	Male *vs* female	6.6 *vs* 4.1	0.24
Age	Above *vs* below median	7.1 *vs* 3.7	0.27
ECOG	0 *vs* 1–2	6.6 *vs* 3.7	0.51
Immunotherapy	With *vs* without prior immunotherapy	7.5 *vs* 3.5	0.34
MSKCC class	MSKCC good *vs* intermediate	4.7 *vs* 5.1	0.35
Heng score	Heng score good *vs* intermediate *vs* poor	4.8 *vs* 5.1	0.51
First-line sorafenib	First-line sorafenib *vs* others	11.3 *vs* 3.7	0.036
Tumour remission first line	Tumour remission *vs* others	4.8 *vs* 6.6	0.58
Second-line TKI before everolimus	Second-line TKI *vs* others	5.1 *vs* 6.6	0.84
Responder to everolimus treatment	Responder *vs* non-responder	7.2 *vs* 4.8	0.61
Interval between TKI and treatment after everolimus	> 6 Months *vs* <6 months interval	6.6 *vs* 5.5	0.68

Abbreviations: ECOG=Eastern Cooperative Oncology Group; MSKCC=Memorial Sloan–Kettering Cancer Centre; PFS=progression-free survival; TKI=tyrosine kinase inhibitor; VEGF=vascular endothelial growth factor.

**Table 3:** Univariable analyses for overall survival from first-line VEGF-targeted therapy

**Independent variables**		**Overall survival (in months)**	***P*-value**
Sex	Male *vs* female	53.3 *vs* 33.5	0.16
Age	Above *vs* below median	43.1 *vs* 31.2	0.21
ECOG	0 *vs* 1–2	43.1 *vs* 31.2	0.42
Immunotherapy	With *vs* without prior immunotherapy	43.1 *vs* 29.8	0.10
MSKCC class	MSKCC good *vs* intermediate	53.4 *vs* 31.8	0.4
Heng score	Heng score good *vs* intermediate *vs* poor	53.4 *vs* 31.8	0.39
First-line sorafenib	First-line sorafenib *vs* others	Not reached *vs* 36.9	0.11
Tumour remission first line	Tumour remission *vs* others	43.1 *vs* 36.9	0.23
Second-line TKI before everolimus	Second-line TKI *vs* others	37.3 *vs* 36.9	0.60
PFS first-line VEGF treatment	Above *vs* below 6 months	53.4 *vs* 19.3	<0.001
Responder to everolimus treatment	Responder *vs* non-responder	36.9 *vs* 43.1	0.70
Interval between TKI and treatment after everolimus	> 6 Months *vs* <6 months interval	36.9 *vs* 37.3	0.43
Liver metastases	With *vs* without liver metastases	31.8 *vs* 46.8	0.18
Bone metastases	With *vs* without bone metastases	29.8 *vs* 46.8	0.22

Abbreviations: ECOG=Eastern Cooperative Oncology Group; MSKCC=Memorial Sloan–Kettering Cancer Centre; PFS=progression-free survival; TKI=tyrosine kinase inhibitor; VEGF=vascular endothelial growth factor.

**Table 4:** Multivariate analyses for PFS of subsequent VEGF-targeted therapy after failure of everolimus

**Independent variables**		**Significance**	**HR**	**95% CI**
First-line sorafenib	First-line sorafenib *vs* others	0.080	0.239	0.048–1.189
Immunotherapy	With *vs* without prior immunotherapy	0.143	0.466	0.168–1.295
Sex	Male *vs* female	0.867	1.100	0.361–3.354
MSKCC class	MSKCC good *vs* intermediate	0.872	1.101	0.343–3.538

Abbreviations: CI=confidence interval; HR=hazards ratio; MSKCC=Memorial Sloan–Kettering Cancer Centre; PFS=progression-free survival; VEGF=vascular endothelial growth factor.

**Table 5:** Multivariate analyses for overall survival in patients with subsequent therapy of everolimus-resistant disease

**Independent variables**		**Significance**	**HR**	**95% CI**
Sex	Male *vs* female	0.145	2.536	0.725–8.872
Immunotherapy	With *vs* without prior immunotherapy	0.125	0.292	0.061–1.409
First-line sorafenib	First-line sorafenib *vs* others	0.658	1.472	0.266–8.164
PFS first-line VEGF treatment	Below *vs* above 6 months	0.002	0.080	0.017–0.383
Interval between TKI and treatment after everolimus	> 6 Months *vs* <6 months interval	0.287	2.224	0.510–9.700
MSKCC class	MSKCC good *vs* intermediate	0.323	0.471	0.106–2.098

Abbreviations: CI=confidence interval; HR=hazards ratio; MSKCC=Memorial Sloan–Kettering Cancer Centre; PFS=progression-free survival; TKI=tyrosine kinase inhibitor; VEGF=vascular endothelial growth factor.
